# The abscopal effects of sonodynamic therapy in cancer

**DOI:** 10.1038/s41416-024-02898-y

**Published:** 2024-11-13

**Authors:** Victoria G. Collins, Dana Hutton, Kismet Hossain-Ibrahim, James Joseph, Sourav Banerjee

**Affiliations:** 1 Department of Neurosurgery, Ninewells Hospital, Dundee, UK; 2 Department of Neurosurgery, Royal Infirmary of Edinburgh, Edinburgh, UK; 3 The Newcastle Upon Tyne Hospitals NHS Foundation Trust, Newcastle Upon Tyne, UK; 4 Department of Biomedical Engineering, School of Science and Engineering, University of Dundee, Dundee, UK; 5 Division of Cancer Research, School of Medicine, University of Dundee, Dundee, UK

**Keywords:** Cancer, Biophysical methods

## Abstract

The abscopal effect is a phenomenon wherein localised therapy on the primary tumour leads to regression of distal metastatic growths. Interestingly, various pre-clinical studies utilising sonodynamic therapy (SDT) have reported significant abscopal effects, however, the mechanism remains largely enigmatic. SDT is an emerging non-invasive cancer treatment that uses focussed ultrasound (FUS) and a sonosensitiser to induce tumour cell death. To expand our understanding of abscopal effects of SDT, we have summarised the preclinical studies that have found SDT-induced abscopal responses across various cancer models, using diverse combination strategies with nanomaterials, microbubbles, chemotherapy, and immune checkpoint inhibitors. Additionally, we shed light on the molecular and immunological mechanisms underpinning SDT-induced primary and metastatic tumour cell death, as well as the role and efficacy of different sonosensitisers. Notably, the observed abscopal effects underscore the need for continued investigation into the SDT-induced ‘vaccine-effect’ as a potential strategy for enhancing systemic anti-tumour immunity and combating metastatic disease. The results of the first SDT human clinical trials are much awaited and are hoped to enable the further evaluation of the safety and efficacy of SDT, paving the way for future studies specifically designed to explore the potential of translating SDT-induced abscopal effects into clinical reality.

## Introduction

Sonodynamic therapy (SDT) is an emerging cancer therapy that uses the interaction between low intensity ultrasound and a chemical agent (termed a sonosensitiser) to produce reactive oxygen species (ROS) which triggers cancer cell death [1, 2]. It has gained attention over recent years as a promising non-invasive cancer treatment modality with the ability to target deep tumours with limited side effects [3–5]. An intriguing and unexpected observation during application of SDT has been the abscopal or ‘vaccine-like’ effect of the intervention. The abscopal effect is a phenomenon where localised therapy causes the regression of distant secondary/metastatic tumours. While the abscopal effect induced by radiation therapy has been studied extensively, the ability of SDT to generate an abscopal effect is largely unexplored [6, 7]. Preclinical evidence hints at its immunostimulatory potential, yet definitive clinical cases demonstrating SDT-driven abscopal effects in humans have not yet been recorded. This review looks to examine the mechanisms of SDT and its potential to cause an abscopal effect. We critically examine in vitro, preclinical, and clinical evidence for the abscopal effect in SDT, identifying opportunities and challenges for future research.

### Sonodynamic therapy as an upcoming therapeutic intervention

SDT can be traced back to the development of photodynamic therapy (PDT) over 30 years ago. PDT utilises a photosensitising agent, selectively absorbed by malignant cells, which is activated by specific wavelengths of light to induce localised cell death [8]. The mechanism of cell death via PDT is debated although the overarching idea is, like SDT, through PDT-induced ROS. Activated photosensitisers release singlet oxygen which affects mitochondrial function, oxidation of pro-apoptotic protein Bcl2 and consequential caspase activation leading to apoptosis [9, 10]. Although effective in vitro, PDT suffers from limited tissue penetration which restricts the efficacy of PDT for targeting deeper tissue tumours [11]. Hence PDT finds its primary clinical relevance as a dermatological intervention. SDT, by comparison, uses low intensity focussed ultrasound (LIFU) and sonosensitisers to target tumours [2]. Compared to light, LIFU has improved tissue penetration and fewer off-target effects and hence SDT has been piped as a deep tissue non-invasive intervention although further preclinical and clinical understandings are essential [2, 12]. Intriguingly, SDT can have functions beyond a direct therapeutic intervention. At lower ultrasound intensities, SDT can also enhance drug delivery. Using microbubbles as transport vehicles, focussed SDT improves tumour-specific chemotherapy drug delivery [13]. This targeted approach has the potential to reduce dose-limiting side effects associated with conventional chemotherapy [14]. Furthermore, SDT has shown potential to overcome the limitations of conventional therapies for solid tumours including pancreatic, breast, lung, prostate, and liver cancers [15–20].

### Mechanisms of cell death in SDT

SDT has proven to be effective in both in vitro and in vivo settings, although the mechanisms underlying sonoactivation and cell death are still unclear [2]. There are currently two well-described mechanisms of action proposed. The first mechanism is through direct disruption of the integrity of the targeted cell while the second mechanism is through the indirect activation of apoptosis through the production of ROS [12, 13]. The current understanding posits that these biological consequences of SDT primarily arise from the acoustic cavitation phenomenon induced by the pulsatile LIFU waves, and the interaction with the chosen sonosensitising agent [2, 21].

#### Acoustic cavitation

Acoustic cavitation comes in two forms: stable and inertial. Stable cavitation occurs when ultrasound waves are passed through a liquid medium containing particles of dissolved gas (microbubbles). Ultrasound excitation cause microbubbles to oscillate between an expanded and contracted state [22]. This subsequently causes fluid movement, creating shearing stresses that can induce cellular membrane disruption—sonophoresis. Transient cell membrane damage induced by SDT offers a novel non-invasive approach to drug transport and delivery [4, 23]. Inertial cavitation, on the other hand, occurs when microbubbles reach a critical size and violent forceful collapse occurs. This collapse generates high-pressure, high-temperature shock waves causing mechanical cell damage, and chemical reactions that potentially activates sonosensitisers [24]. Additionally, the forceful collapse can convert ultrasound energy into a flash of light termed sonoluminescence [13, 23]. Sonoluminescence is another proposed mechanism for sonosensitiser activation.

#### Generation of ROS

As stated previously, ROS generation is crucial for tumour cell death in SDT. One proposed mechanism is through the direct mechanical and thermal stress caused by cavitation [25]. Heat and shearing forces can induce molecular pyrolysis and the breakdown process triggers release of free radicals that react with endogenous substrates to generate ROS [25]. ROS are also produced when sonosensitisers are activated by sonoluminescence. On excitation, the sonosensitiser transfers energy to surrounding oxygen, resulting in cytotoxic oxidative reactions within tumour cells [13]. SDT can therefore be limited by hypoxic environments [13]. Additionally, activated sonosensitisers may destabilise the cell membrane, increasing susceptibility to the local shearing forces of ultrasound-induced cavitation [13, 26]. The resulting ROS can effectively damage intracellular DNA and proteins, promote cellular lipid peroxidation, and induce apoptosis of target cells [27].

SDT-mediated death is primarily observed in tumour cells when they preferentially take up sonosensitisers. Sonosensitisers are of various chemical backbones.

### Sonosensitisers

Sonosensitisers play a crucial role in the SDT process by converting ultrasound energy into chemical energy directly generating ROS. In a narrow sense, the effectiveness of SDT is heavily influenced by the performance of the sonosensitiser and targeted uptake of sonosensitisers make SDT a selective tumour treatment approach [28]. There are two types of sonosensitiser: organic and inorganic. Organic sonosensitisers show good biodegradability, potent ROS generation, and a fixed chemical structure compared to inorganic sonosensitisers. They also demonstrate poorly targeted accumulation, weak solubility in water, and have extreme phototoxicity. Advancements in targeted delivery methods aim to optimise tumour tissue accumulation, contributing to the ongoing refinement of organic sonosensitisers for more effective SDT [29]. Inorganic nanostructures have been similarly investigated and have achieved promising results over the past few years [30]. These include carbon-based, silicon-based, and noble metal sonosensitisers. This section will review common organic and inorganic sonosensitisers with a focus on safety, clinical usage, and targeted outcomes. Table 1 summarises the various types of sonosensitisers reported for SDT applications.

**Table 1:** Overview of sonosensitisers.

Sonosensitiser type	Subtype	Pros	Cons
Organic	Porphyrins (PpIX, hematoporphyrin, etc.)	Adjustable photoelectric properties	Chemically unstable, toxic to skin, low specificity, rapid metabolism
	5-Aminolevulinic Acid (5-ALA)	Better bioavailability, preferential uptake by cancer cells, especially gliomas	Requires conversion to PpIX for ROS generation, limited to cells with active haem biosynthesis pathway
	Xanthenes (Rose Bengal)	Significant cytotoxic effects with ultrasound, selective anti-tumour effects	Limited studies on long-term effects
Inorganic	Carbon-based materials	Increasingly recognised for use in PDT and photothermal therapy	Low charge separation rate, weak solubility
	Noble metal-based (TiO_2_, Mn, Pt, Ag, Au)	Adaptable properties, high stability	Suboptimal tumour accumulation, potential for increased hypoxia and GSH production in TME
	Silicon-based (nanoparticles, nanowires)	Highly biodegradable, biocompatible, low cytotoxicity	Limited studies on long-term effects

#### Organic sonosensitisers

Porphyrin-based sonosensitisers are similar in carbon core structure and generation of ^1^O_2_ [29]. A key benefit of porphyrin-based sonosensitisers is that their photoelectric properties can be adjusted depending on clinical indication or target by the coordination of the metals held within the porphyrin ring [31]. Commonly used examples include protoporphyrin IX (PpIX), haematoporphyrin, monomethyl ether, and photofrin. However, porphyrin-based sonosensitisers have associated side effects; they are often chemically unstable and toxic to the skin. Porphyrin-based agents have also been reported to have low-specificity and rapid metabolism—limiting their use in SDT and clinical application [31].

5-aminolevulinic acid (5-ALA) is an endogenous, porphyrin-based molecule that is formed in the mitochondria from succinyl-CoA and glycine. 5-ALA is a precursor of PpIX and is implicated in the biological synthesis of haem. PpIX is formed through the conjugation of eight ALA molecules and is metabolised into haem via the rate-limiting enzyme, ferrochelatase. When 5-ALA is given exogenously (increasing its level above the biological baseline), abundantly produced PpIX cannot be quickly converted into haem, and therefore accumulates within cells [32]. The stepwise metabolisation and accumulation of this pro-drug results in a better bioavailability of 5-ALA in comparison to the porphyrin-based agents mentioned above. In normal cellular conditions, 5-ALA does not produce ROS. However, when exogenous 5-ALA is administered and exposed to focussed ultrasound (FUS), the accumulated PpIX metabolite is activated by the FUS to produce ROS. This, in turn, can trigger cell death within targeted cells through the mitochondrial apoptotic pathway [33].

5-ALA is also preferentially taken up by cancer cells and has shown to be particularly sensitive for high-grade glioma cells and is the most studied sonosensitiser for glioma-SDT [34, 35]. The exact mechanism of glioma-specific uptake of 5-ALA is unclear but there are multiple current theories. Firstly, there is varied activity of ferrochelatase within tumour cells [36]. Additionally, the uptake and accumulation PpIX could also be influenced by impairment of the blood-brain barrier commonly seen with high-grade gliomas [33]. More recent studies with advanced imaging techniques have found significant PpIX accumulation in tumour-associated macrophages and extracellular spaces [37].

Xanthenes are a class of dyes that have demonstrated potential in SDT. Notably, Rose Bengal (RB) (disodium 4,5,6,7-tetrachloro-2ʹ,4ʹ,5ʹ,7ʹ-tetraiodofluorescein), a type of xanthene dye, has shown a significant increase in cytotoxic effects combined with ultrasonic irradiation [18, 38]. RB-mediated SDT has also proven in a rat intracranial glioma model to exert selective anti-tumoral effects without causing any damage to surrounding normal brain tissue [18].

#### Inorganic sonosensitisers

Carbon-based nanomaterials, while increasingly recognised for their roles in PDT and photothermal therapy, face challenges as sonosensitisers in SDT due to their low charge separation rate and weak solubility [39, 40]. Techniques like combination therapy, grafting water-soluble molecules, and improving tumour site accumulation are being investigated to overcome these obstacles. However, significant further research is necessary to achieve better SDT output with carbon-based nanomaterials [30].

##### Noble metal-based sonosensitisers

Examples of noble metal-based sonosensitisers include titanium oxide (TiO_2_)-, manganese (Mn)-, platinum (Pt)-, silver (Ag)-, and gold (Au)-based nanosystems. These metal complexes offer adaptable physiochemical properties - including the ability to minimise the energy gap between the highest occupied molecular orbital and lowest occupied molecular orbital, and thus increasing their ’sonosensivity’ [41]. Metal-based agents show great promise as sonosensitisers as they have inherent acoustic cavitation qualities and high stability, as well as water solubility, and biocompatibility on PEGylation and modification with hydrophilic monomers [30, 42]. However, these agents alone are limited by suboptimal accumulation in tumour regions due to easy capture by the reticuloendothelial system [43]. Additionally, noble metal-based agents have been associated with increased hypoxia and overproduction of glutathione (GSH) in the tumour microenvironment (TME). Excess GSH significantly compromises SDT therapeutic efficiency by continuously scavenging the newly SDT-generated ROS [30]. To overcome these barriers, they are being combined with other materials that improves bioavailability, and augments ROS production through TME remodelling [30].

##### Silicon-based sonosensitisers

Silicon-based materials are highly biodegradable, biocompatible, and associated with a low cytotoxicity. They can also be enhanced with noble metals or biopolymers for use in SDT. Examples of silicon-based sonosensitisers include silicon nanoparticles, nanowires, and nanocrystals [43]. Fabricated silicon nanowires in combination with FUS has shown to decrease the survival rate of targeted cancer cells to approximately 50%, compared to FUS alone [44]. Nanoparticles containing mesoporous silicon (MSNs) are also effective carriers of sonosensitisers. Interfacial nanobubbles (INBs) and various sonosensitisers can be trapped within the mesoporous cavity of the MSN and offers longitudinal stability. On exposure to FUS, these INBs produce ROS secondary to molecular pyrolysis of water during cavitation [43].

## Mechanisms of SDT-induced abscopal effect

Coined in 1953, the abscopal effect describes the rare phenomenon of distant tumour regression induced by localised therapy [45]. It was initially observed with radiotherapy and later with immunotherapy [46, 47]. Subsequent research has revealed the involvement of the immune system in the generation of the abscopal effect. Key reviews on this topic in radiotherapy include Rodriguez-Ruiz et al. [48] and Grass et al. [48, 49]. This mechanism is reviewed below.

Immunogenic cell death (ICD) is any form of regulated cell death that leads to the activation of an immune response by the release or exposure of danger-associated molecular patterns (DAMPs) and tumour-associated antigens (TAAs) [50–52]. Tumours possess sophisticated mechanisms to evade immune detection involving reduced immune recognition through absence of strong tumour antigens and downregulation of MHC molecules [53–55]. Additionally, they develop an immunosuppressive tumour microenvironment by the generation of immunosuppressive factors such as TGF-β and VEGF [53–55]. The generation of large amounts of DAMPs by SDT-induced ICD increases the likelihood of an effective immune response [56]. Depending on the exact mechanism of cell death, different types and volumes of DAMPs are generated. These include calreticulin exposure, phosphatidylserine (PS) externalisation, ATP release, and passive high motility group box 1 (HMGB1) protein release [50, 51]. Once on the cell surface, calreticulin and PS increase the antigenicity of the dying cancer cells by driving a pro-phagocytic response [57]. Extracellular ATP also acts as a ‘find me’ signal that recruits monocytes as well as activating purinergic P2X_7_ receptors from dendritic cells which leads to the subsequent activation of the NOD-like receptor family, pyrin domain containing-3 protein (NLRP3)-dependent caspase-1 activation complex (‘inflammasome’) and ultimately driving the secretion of IL-1β [52, 58]. Extracellular HMGB1 is generally agreed to induce inflammation by binding to pattern recognition receptors (PRRs) including Toll-like receptor 4 (TLR4), Toll-like receptor 2 (TLR2), and the receptor for advanced glycosylation end products (RAGE) of immune cells in the tissue surrounding the dying cells and generating further strong pro-inflammatory cytokine release as well as stimulation of antigen-presenting functions within dendritic cells (DCs) [51, 52, 59, 60].

**Fig. 1:**
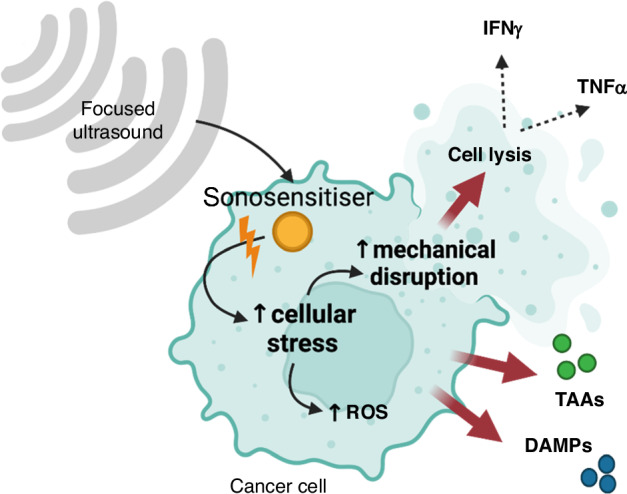
Effect of sonosensitiser activation. Through various mechanisms, low intensity focussed ultrasound activates sonosensitisers causing cellular stress in cancer cells through increased mechanical disruption and generation of reactive oxygen species (ROS). Mechanical disruption itself can precipitate cell lysis and in response, release of interferon-gamma (IFN-γ) and tumour necrosis factor alpha (TNF-α). Build-up of intracellular ROS can trigger immunogenic cell death leading to release of tumour associated antigens (TAAs) and damage-associated molecular patterns (DAMPs).

**Fig. 2:**
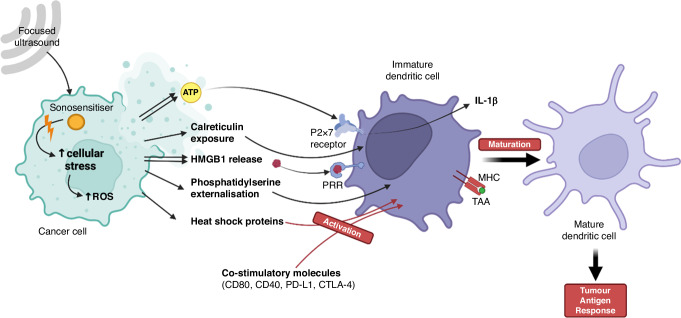
Process of immunogenic cell death. Once immunogenic cell death is activated, damage-associated molecular patterns (DAMPs) are released. Calreticulin exposure and externalisation of phosphatidylserine on the surface of the stressed cell act as to signal local immature dendritic cells. High motility group box 1 (HMGB1) protein release binds to pattern recognition receptors (PRRs) to increase inflammation. Extracellular ATP simultaneously acts as a ‘find-me’ signal itself and also activates purinergic P2X_7_ receptors on dendritic cells. Heat shock proteins generated secondary to cellular stress also contribute to the activation of dendritic cells. Once the immature dendritic cells are activated and phagocytose cellular fragments, tumour associated antigens (TAAs) become present on the major histocompatibility complexes leading to antigen presentation, maturation and tumour antigen response.

Another important category of DAMPs in SDT-induced cell death are heat shock proteins (HSPs). HSPs are normally intracellular anti-apoptotic chaperones generated in reaction to cellular stress to the endoplasmic reticulum that act to remodel proteins [61, 62]. In ICD, however, intracellular HSPs can be shifted onto the cellular membrane. In particular, HSP70 and HSP90 in the dying cells become exposed on the surface membrane, interact with receptors on immature dendritic cells (iDCs), CD40 and CD91 respectively, and contribute to the activation of DCs [62–64]. These processes are shown in Fig. 1 and Fig. 2.

Entire cells or cell fragments from tumour cells that have undergone a form of programmed cell death are then taken up by antigen-presenting cells (APCs). This SDT-induced tumour antigen shedding results in phagocytosed fragments being digested into smaller peptides that contain the epitopes then presented by the major histocompability complex (MHCs) on the surface of APCs. The DAMPs released during the initiation of ICD drive increased recruitment of cells, increased recognition and phagocytosis. Additionally, they contribute to the upregulation of co-stimulatory molecules (such as CD80, CD40, PD-1, and CTLA-4) which lead to the activation of the iDCs by intracellular upregulation of the expression of MHC class II molecules [65, 66]. These iDCs then undergo transformation into mature dendritic cells (mDCs) via a complex biochemical pathway involving the complete uptake of antigens and initiation of their migration to adjacent lymph nodes or the spleen (secondary lymphoid organs) to continue immune activation [65–67]. A fully mature dendritic cell is then able to activate the adaptive immune system.

**Fig. 3:**
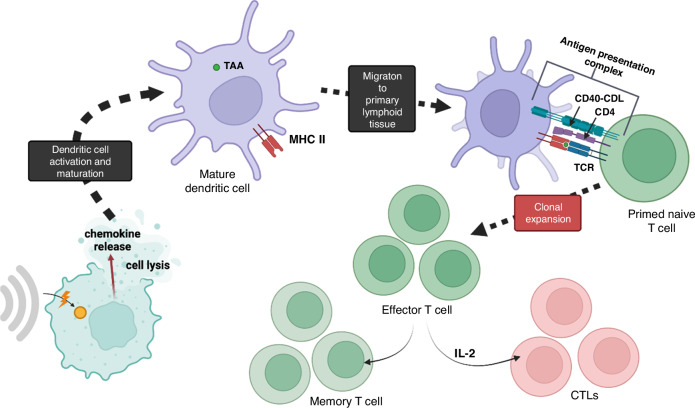
Immune cascade. The activation and maturation of dendritic cells are the first step in the immune cascade that causes the abscopal effect. Immature dendritic cells (iDCs) migrate to primary lymphoid organs where they mature. Following this, they prime naive T cells and trigger clonal expansion.

Upon reaching a secondary lymphoid organ, antigen-loaded mDCs encounter naive T cells (Th0) where the tumour antigens of the mDCs bind to the T cell antigen receptor of the Th0. This process triggers clonal expansion and differentiation of Th0 into effector T cells. This begins with the triggering of a cascade of intracellular signalling events orchestrated by cytokines, such as interleukin-2 (IL-2), which promotes proliferation of activated T cells and cell cycle progression into a group of antigen-specific effector T cells, including cytotoxic T lymphocytes (CTLs) [68]. This process also involves stringent selection mechanisms which ensure that only T cells with high-affinity receptors and appropriate co-stimulation undergo clonal expansion [68]. Additionally, some activated T cells differentiate into memory T cells, ensuring the establishment of immunological memory towards the tumour neoantigen [68]. This process is summarised in Fig. 3.

**Fig. 4:**
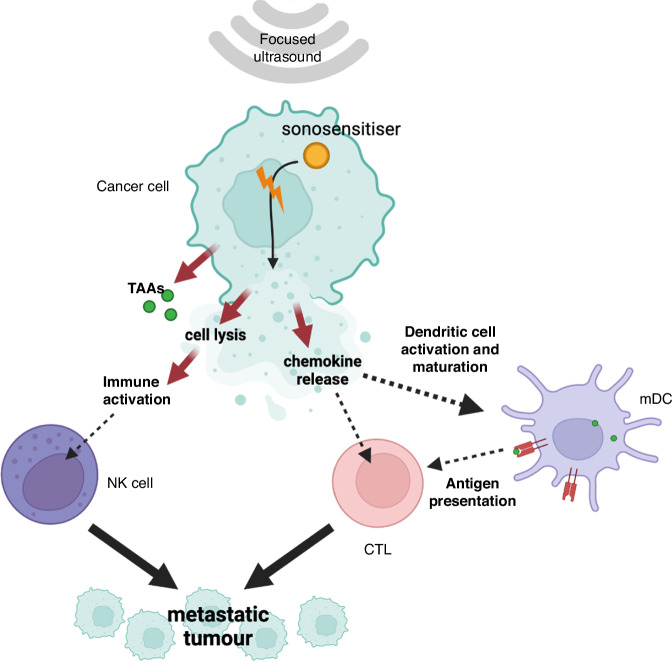
Immune stimulation by SDT. The ultrasound activated sonosensitiser triggers cell death both from mechanical disruption and generation of ROS. This triggers multiple pathways of immune cell activation to generate a coordinated and targeted immune response sensitive to both the primary tumour and metastatic lesions.

CTLs are then disseminated throughout the body and able to reach metastatic tumour sites. Here, they recognise tumour cells expressing cognate antigens and initiate a cascade of effector functions, including the release of cytotoxic granules containing perforin and granzymes, as well as the expression of death ligands such as Fas ligand (FasL) and tumour necrosis factor-related apoptosis-inducing ligand (TRAIL) [69–71]. These effector molecules then induce apoptosis in target tumour cells. CTLs also produce cytokines such as interferon-gamma (IFN-γ) and tumour necrosis factor-alpha (TNF-α), which further promote antitumour immune responses by enhancing the activation of other immune cells and modulating the tumour microenvironment [69, 72]. Additionally, the recognition and killing of tumour cells by CTLs lead to the release of additional TAAs and DAMPs, which further amplify the immune response and facilitate the recruitment and activation of additional effector cells, including macrophages and natural killer (NK) cells [50, 52]. This coordinated immune response (Fig. 4) ultimately results in the regression of not only the primary treated tumour but also distant metastatic lesions, thereby eliciting the abscopal effect. This contribution to systemic antitumour immunity is SDT – induced vaccine effect.

### Conversion of immunologically cold tumours

Glioblastoma multiforme (GBM) has an immunologically “cold” TME created by a lack of strong TAAs, secretion of immunosuppressive factors, and occasionally downregulated MHCs [73–75]. This is defined by the degree of T cell infiltration. In comparison, a “hot” tumour is T cell-infiltrated and susceptible to T cell-mediated attack [76, 77]. However, GBMs also have an immunosuppressed myeloid landscape that works to control immune response while allowing tumour cell proliferation [78–81]. These mechanisms mean that immunotherapy has little effect on GBM [82].

One study investigating this showed that a combined Rutherrin-PDT increased CD8 + T-cells in a rat glioma model [83]. The immune-modulating effect on the TME would therefore convert the initially cold tumour to a hot tumour that would have higher susceptibility to immunological treatments. The identification of the exact immunological response to SDT and subsequent effects of this on the TME is therefore needed to further assess the clinical role of SDT in cancer treatment both as an individual and combined therapy [84, 85].

### SDT in brain cancers

Beyond this, gliomas represent a category of primary malignant brain tumours characterised by their genetic heterogeneity, as well as their inherently aggressive and invasive behaviour. Even with optimal treatment, often recur locally with an associated poor prognosis [86]. Alongside combatting chemo- and immuno-escape in immunological conversion, the tissue depth penetration is also advantageous for deep unresectable brain tumours [3, 87, 88]. Additionally, the specificity for glioma cells without damaging healthy tissue decreases post-procedural morbidity with this preservation of surrounding normal brain tissue confirmed in in vivo studies [12, 34, 88]. SDT with 5-ALA is being investigated in an ongoing phase 1–2 study of diffuse intrinsic pontine glioma (DIPG)—an aggressive paediatric high-grade glioma [88]. These tumours are located within the brainstem and cannot be fully resected surgically. Chemotherapy shows limited effectiveness, and targeted radiotherapy is still largely palliative [89, 90]. Therefore, a minimally invasive glioma-specific therapy such as SDT may be an option in the future for these children.

## Preclinical evidence of the abscopal effects of SDT in cancer

This section overviews the preclinical evidence of the abscopal effects of SDT in cancer—with simple sonosensitisers, more novel methods of sonosensitiser delivery (nanoparticles and microbubbles), and in conjunction with chemotherapy and immunomodulating agents. Most studies in the literature are not specifically targeted at the abscopal effect, but a small number of pre-clinical studies primarily aimed at the study of novel sonosensitiser models mention findings demonstrating the ability of SDT to induce distant tumour effects in metastatic cancer mouse models (Table 2).

**Table 2:** Summary of preclinical evidence of the abscopal effect of SDT.

Group	Cancer cell type	Sonosensitiser	Model used	Mouse type	US power density	US frequency	Duty cycle	Pulse repetition frequency	Exposure time (*n* = number of exposures)	Adjunct(s) used
Zhang et al. [91]	Liver	HPD	HPB + FUS	Immunocompromised and immunocompetent	1 W/cm^2^	1 MHz	50%	Unspecified	2 h (*n* = 3)	-
Chen et al. [95]	Breast	Protoporphyrin	Nanosonosensitiser manganese-protoporphyrin within folate liposomes + SDT	Immunocompetent	2.0 W/cm^2^	1.0 MHz	50%	Unspecified	5 min (*n* = 1)	-
Fu et al. [99]	Breast	PEGylated CoFe^2^O_4_ nanoflowers (CFP)	CFP + CDT + SDT	Immunocompetent	0.5 W/cm^2^	1.0 MHz	20%	Unspecified	3 min (*n* = 2)	a-PD-L1
Nesbitt et al. [47]	Pancreatic	Rose Bengal	MBs with Rose Bengal + SDT	Immunocompetent	3.5 W/cm^2^	1.0 MHz	30%	100 Hz	3.5 min (n = 2)	a-PD-L1
Nicholas et al. [93]	Pancreatic	Rose Bengal	NPs with Rose Bengal + SDT	Immunocompromised	3 W/cm^2^	1 MHz	30%	100 Hz	3.5 min (*n* = 1)	-
Zhan et al. [97]	Ovarian	Mn-TCPP	Nanoplatform with Mn-MOF and CpG, lined with B16 cells overexpressing OVA + SDT	Immunocompetent	1 W/cm^2^	1 MHz	50%	Unspecified	10 min (*n* = 3)	a-PD-L1
Zheng et al. [100]	Ovarian	ICG	NPs with ICG + oxaliplatin + PSDT	Immunocompetent	1 W/cm^2^	Unspecified	Unspecified	Unspecified	1 min (*n* = 1)	Oxaliplatin
Hadi et al. [96]	Pancreatic	Hematoporphyrin	NPs with hematoporphyrin + SDT	Immunocompetent	3.5 W/cm^2^	1.0 MHz	50%	100 Hz	3.5 min (n = 1)	-
Yang et al. [98]	Colorectal	Titanium disulfide	PEG-modified manganese-doping titanium disulphide NSs + gas therapy + SDT	Immunocompetent	3 W/cm^2^	30 kHz	Unspecified	Unspecified	10 min (*n* = 3)	a-PD-L1
Qiao et al. [94]	Breast	BP	PEG-modified BP NSs + SDT	Immunocompetent	0.75 W/cm^2^	1 MHz	30%	Unspecified	10 min (*n* = 5)	-
Wang et al. [92]	Breast	MoOX	PEG-modified MoOX NPs + SDT	Unspecified	3 W/cm^2^	40 kHz	Unspecified	Unspecified	15 min (*n* = 3)	a-CTLA-4

There is limited comparability of these studies due to the range of different methodologies and sonosensitisers used. However, most use bilateral mouse tumour models with injection of the selected cancer cell line being studied into both the right and left flanks of mice prior to exposure to the sonosensitising system, SDT and/or any adjuvants being used. SDT is applied only to one flank (the primary tumour). If any tumour regression is seen in the contralateral tumour (the off-target tumour), this models the abscopal effect – and represents that there is a degree of immune recognition of a tumour outside the sonicated field.

### SDT on its own

**Fig. 5:**
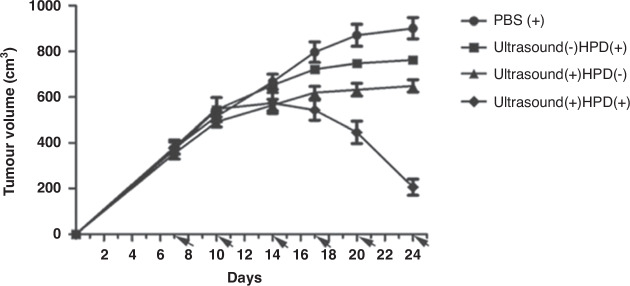
Tumour growth inhibition after 6 SDT cycles. HiPorfin (HPD) combined with ultrasound irradiation effectively inhibited tumour growth in bilateral liver cancer mouse models (*P* < 0.001, vs control). Data expressed as means ± SD (*n* = 10). Figure from Zhang et al. [91] under Creative Commons license.

A recent study has shown SDT to induce abscopal effects in bilateral orthogonal liver cancer mouse models [91]. The research group used FUS (frequency = 1 MHz, power intensity = 1.0 W/cm^2^, duty cycle (DC) = 50%, exposure time = 2 h per cycle (*n* = 3)) in combination with the sonosensitiser HiPorfin (HPD). HPD alone did not significantly inhibit distant tumour growth compared to the control (tumours injected with PBS). However, the combination of HPD + FUS caused a significant delay in distant tumour growth (*P* < 0.001 vs control)—with 20% of the mice treated with HPD + FUS achieving complete abscopal responses [91]. Furthermore, re-inoculation of liver cancer cells 30 days later into the flanks of the 20% of mice who achieved a cure with SDT, resulted in no new cancer growth (compared to primary and metastatic tumour growth in naïve control mice and death within 60 days) (Fig. 5). This suggests that SDT has the potential to be developed into a novel cancer vaccine therapy.

### Nanomaterials and SDT

Nanomaterials, including nanoparticles and nanosheets, have been used by multiple groups as a method of delivering sonosensitisers and anti-tumour therapies to enhance and work in synergy with SDT respectively [47, 92]. Many nanomaterials specifically are adapted and engineered to counteract the barrier that tumour hypoxia poses.

pH-sensitive polymethacrylate-coated CaO_2_ nanoparticles containing RB (RBcNPs) are nanoparticles capable of transiently alleviating tumour hypoxia through in situ generation of oxygen in response to the low pH in hypoxic tumours [93]. The RBcNPs were injected into bilateral murine tumour models of pancreatic cancer. Ultrasound was delivered within 5 min of administering RBcNPs (1 MHz, 3 W/cm^2^; DC = 30%; pulse repetition frequency (PRF) = 100 Hz for 3.5 min (*n* = 1)). RBcNP-mediated SDT delivered a potent abscopal effect accompanied by an increase in tumour cytotoxic T cells and a decrease in immunosuppressive tumour regulatory T-cells in both target and off-target tumours [93].

Black phosphorus nanosheets (BPNS) that are poly(ethylene glycol) (PEG)-modified and loaded with 3-Pyridinyl)-1-(4-pyridinyl)-2-propen-1-one (3PO) (termed BO) have also been developed in attempt to boost the efficacy of SDT [94]. Under FUS, BO can produce ROS leading to further hypoxia and nutrient block. 3PO also inhibits tumour glycolysis and lactate accumulation. Both can cut off the source of lactic acid and achieve anti-tumour starvation therapy. This lactate depletion in combination with SDT facilitates DC maturation, antigen presentation, and ultimately anti-tumour immunity and inhibition of distant tumour growth [94]. The above mechanism for the abscopal effect of SDT with BO was proven in bilateral breast cancer tumour mouse models. Tumour-bearing mice were divided randomly into 5 groups (1) PBS; (2) US; (3) BO; (4) BP + US (5) BO + US. In the relevant groups, SDT was carried out 12 h after of intravenous injection (0.75 W/cm^2^, 1 MHz, DC = 30%, 10 min), every 4 days for 13 days. The combined treatment of BO + SDT profoundly reduced both the treated primary and untreated distant tumour volumes compared to control. As well as this profound immune response, the combination therapy achieved good biological safety [94].

The use of a multifunctional nano-sonosensitiser system (FA-MnPs) to activate an SDT-mediated response in bilateral triple-negative breast cancer mouse models has also shown the abscopal effect [95]. The designed system encapsulates manganese-protoporphyrin (MnP) into folate-liposomes (correlating to folic acid (FA) receptors overexpressed in many cancers) and incorporates the photosensitiser protoporphyrin IX chloride. A single exposure of FUS was delivered to the primary tumours at 1 MHz over 5 mins (2 W/cm^2^, DC = 50%). The FA-MnPs+US group is broken down into two subgroups: the FA-MnPs+US(s) group which only treated the right-sided tumour and the FA-MnPs+US(d) group which used depth-penetrating ultrasound from the right tumour to the left. Compared with the rapid growth in the control (PBS) group, PBS + US and FA-MNPs group, both primary and distant growth was most effectively inhibited in the FA-MnPs+US groups with (s) group showing abscopal distant tumour shrinkage (*p* < 0.01 for primary growth in both subgroups, p < 0.05 in (s) distant growth, *p* < 0.001 in (d) distant growth) [95].

A nanoparticulate formation (HPNP) based on a cathepsin B-degradable glutamate-tyrosine co-polymer that carries hematoporphyrin was showed the abscopal effect when used for SDT-based treatment of bilateral pancreatic tumours in immunocompetent (KPC) mice [96]. The animals were in the following treatment groups: no treatment, HPNP, US and HPNP + US. Target tumours were exposed to a FUS for 3.5 mins (3.5 W/cm^2^, 1 MHz, PRF = 100 Hz, DC = 50%) SDT treatment in combination with HPNP resulted in 21% and 27% reductions in target and off-target tumour volumes within 24 h, respectively—illustrating both direct tumoral and abscopal effects [96].

### Microbubbles and SDT

Microbubble mediated SDT has been found to generate an abscopal response in a bilateral pancreatic cancer mouse model [47]. This group also used RB as a sonosensitising agent – but instead used a systemic microbubble-RB conjugate (MB-RB). Ultrasound irradiation (3.5 W/cm^2^, 1 MHz, DC = 30%, PRF = 100 Hz, 3.5 mins) was applied to the target tumour in two exposures - during, and immediately after MB-RB administration. At 11 days post-SDT treatment, irradiated mice were observed to have an average decrease in tumour volume of 287% (average volume between both bilateral tumours) [47].

### Combined SDT with an Immune-Checkpoint Inhibitor (ICI)

Multiple studies suggest that both the primary effect and abscopal effect of SDT is significantly enhanced when combined with an adjuvant immunotherapeutic agent such as anti-PD-L1 [47]. Additionally, mice receiving SDT+anti-PD-L1 therapy to their primary tumour have been observed to have significantly higher levels of infiltrating CD4+ and CD8 + T-cells in any residual off-target tumour tissue, compared to those who did not receive SDT+anti-PD-L1 therapy. These results suggest SDT treatment elicits an adaptive immune response that is further potentiated by anti-PD-L1 ICI therapy in this model of pancreatic cancer [47].

Research studies also showed the immune modulatory effects of SDT in combination with anti-PD-L1 therapy where an SDT-mediated nanovaccine model for potentiating anti-PD-L1 antibody therapy in malignant melanoma was created [97]. The nanoplatform was synthesised from binding Mn-MOF with immune adjuvant CpG oligonucleotide, and then coating this system with cell membranes derived from melanoma B16 cells that overexpress ovalbumin (OVA) antigen. Together with CpG, the tumour-associated antigens derived from SDT and OVA elicited a strong tumour-specific immune response [97]. In a bilateral melanoma tumour model (using B16-OVA cells), combination of anti-PD-L1 antibody and Mn-MOF/CpG nanoplatform with US irradiation (1 MHz, 1 W/cm^2^, DC = 50%, for 10 mins (*n* = 3; five-day intervals)) significantly delayed the growth of both irradiated primary tumours and non-irradiated distant tumours. Two of eight mice were tumour free of primary tumours after combination of anti-PD-L1 antibody plus nanoplatform with US irradiation-treated group, resulting in longer survival time. Thus, the nanoplatform showed strong systemic antitumour responses upon FUS and improved the therapeutic effects of anti-PD-L1 antibody [97].

PEG-modified manganese-doping titanium disulphide nanosheets (PEG-MnTiS NSs) have been fabricated as a cascade bioreactor for sequential gas therapy (GT)-enhanced SDT. In this nano-system, titanium disulphide acted as an efficient sonosensitiser. In vitro and in vivo results illustrated outstanding outcomes of sequential GT-SDT alone showing an increase in ROS generation (US parameters: 3 W/cm^2^, 30 kHz, for 10 min (*n* = 3; two-day intervals)) [98]. The responsible group also considered possible immune tolerance and therefore used sequential anti-PD-L1 antibodies. They found that this combination elicited excellent responses on primary and distant tumours compared to that achieved in the control, anti-PD-L1 only, and the MnTiS-PEG + US groups [98].

An abscopal response to SDT+anti-PD-L1 antibody therapy has been reported in a murine mouse cancer model using PEGylated CoFe_2_O_4_ nanoflowers (CFP) – a novel sonosensitiser – to augment combined SDT + chemodynamic therapy (CDT) where in vivo suppression of both primary and distant tumour growth was achieved [99]. BALB/c mice bearing bilateral 4T1 tumours were assigned to four groups (1) saline, (2) US, (3) CFP, (4) CFP + US. On days 1 and 3 post IV injection of CFP, the tumour regions of the relevant groups were exposed to US irradiation (1 MHz, 0.5 W/cm^3^, DC = 20%, for 3 min (*n* = 2; days 1 and 3)). Compared with the saline, CFP resulted in slight tumour inhibition due to the CDT effect, but the best primary tumour elimination was in mice receiving CFP + US. Using a synergy of additional PD-L1 checkpoint blockade achieved the most significant abscopal effect – with treatments of “CFP+anti-PD-L1” and “CFP + US+anti-PD-L1” resulting in an outstanding suppression on primary tumour growth, with a tumour growth inhibition of 46.7% and 66.3%, respectively. In contrast, administration of US+anti-PD-L1 exhibited insignificant inhibitory effect on the growth of both primary and distant tumours [99].

Another ICI + SDT achieving abscopal effects in vivo studies are anti-CTLA-4 antibodies. PEG-modified oxygen-deficient molybdenum oxide (MoOx) nanoparticles have been developed and used in combination with SDT (40 kHz, 3 W/cm^2^, for 15 mins (*n* = 3)) and anti-CTLA-4 in mice bearing bilateral breast cancer tumours [92]. Distant tumour growth from SDT treatment was partially inhibited; however, MoOx-triggered SDT plus anti-CTLA-4 significantly decreased distant tumour growth. Additionally, the survival of these mice was extended 2-fold compared to control and anti-CTLA-4 alone—with no significant side effects. The group also proceeded to perform lung metastasis assays on the 4T1 tumour-bearing mice. This revealed fewer pulmonary lesions to be observed in the MoOx+SDT and MoOx+SDT+anti-CTLA-4 groups (compared to control and anti-CTLA-4 alone) – particularly in the MoOx+SDT+anti-CTLA-4 group [92].

### Combined SDT with chemotherapy

Considering multi-modality approaches, PSDT has been shown to induce systemic antitumour immunity in a bilateral syngeneic ovarian cancer cell mouse model [100]. A study combined PDT and SDT to decrease the dose of sensitiser as well as the amount the energy of ultrasound/light delivered in attempt to lessen potential side effects. A new class of sensitiser that has both the photosensitiser indocyanine green (ICG) (a near-infrared fluorescent contrast agent), and oxaliplatin in their shell with oxygen at its core was used. The nanoparticles were injected at an oxaliplatin dose of 2 mg/kg every five days for four cycles. After 4 h, primary tumours were exposed to 808 nm laser irradiation (1.5 W/cm^2^, 5 mins) combined with a single exposure of US (1 W/cm^2^, for 1 min) to activate the ICG and release oxaliplatin. The combined strategy of nanoparticle-mediated PSDT and oxaliplatin induced immunogenic cell death of the primary tumour by promoting surface exposure of calreticulin and passive release of HMGB1. The nanoparticles also inhibited the growth of both primary and distant tumours. The latter was proposed to be mediated by increased infiltration of cytotoxic T lymphocytes of the distant tumour [100].

### Animal models used in SDT research

Current published works have primarily used immunocompromised mice for tumour modelling. However, this review indicates that most studies showing the abscopal effect have been on immunocompetent mice aligning with the likely immunological underlying mechanism of the abscopal effect. Further work using syngeneic murine models would be the gold standard for immunological studies as a more clinically relevant translational model [101].

## Evidence of the abscopal effect of PDT and SDT in human clinical trials

PDT has previously shown evidence of producing an abscopal effect in the treatment of GBM in humans. Deep-seated GBM was treated with immunophotodynamic therapy (iPDT) with subsequent shrinkage of satellite lesions outside the treatment field [102]. Similar distant tumour shrinkage has been observed in PDT-treated patients both with and without an ICI adjunct [103, 104]. The compelling preclinical efficacy of SDT has led to several clinical trials investigating its use in cancer. SDT has none of the oedema seen in PDT [105, 106]. It is also non-invasive and more easily repeatable than PDT. A phase 1/2a clinical trial of SDT for GBM showed no severe adverse effects (SAEs) and the Washington Children’s Hospital had no SAEs in the clinical trial initial data for SDT in DIPG [88, 107]. However, as the only published studies are in early phases, it is too soon to see any abscopal effects of SDT in a human population.

## SDT and HIFU

High intensity focussed ultrasound (HIFU) is a similar emerging therapeutic modality that uses higher energy ultrasound sonication to cause high temperatures leading to coagulative necrosis of the targeted tissue without the use of a sensitiser [108]. The non-invasive nature of HIFU and precise targeting is otherwise similar to SDT. It has been used in some solid tumours and in targeted drug release [108]. Beyond cancer treatment, it is used in magnetic resonance guided thalamotomy for the treatment of movement disorders [109].

While the abscopal effect of SDT is a relatively new area of investigation, a review of preclinical and clinical evidence indicated that HIFU can induce various immune responses, including increasing the activity of cytotoxic T lymphocytes and natural killer cells [110]. These immune responses are thought to be triggered by the release of tumour antigens and heat shock proteins from the ablated tissue, as well as the creation of an inflammatory environment similar to the proposed mechanism behind the SDT-mediated abscopal effect [110–112]. Further comparison is beyond the scope of this review.

## Conclusions and future directions

SDT is a promising non-invasive and targeted cancer treatment. SDT has shown to have positive effects in human cancer therapy but requires more systematic investigations to establish standardised protocols for sonosensitiser delivery, treatment parameters, and combination therapies. Sonosensitisers and carriers of sonosensitisers also require continued development with a focus in improving tumour targeting and overcoming the challenges due to TME. Combination therapies of SDT and ICI, PSDT, and SDT with chemotherapy also show significant promise as well.

Preclinical evidence shows that SDT can induce an abscopal vaccine effect. This phenomenon suggests the possibility of local targeted therapy having systemic effects in advanced or metastatic cancers. The underlying immunological mechanisms still require further exploration. Identifying the specific immune cell populations and pathways involved in SDT-induced abscopal responses would lay the groundwork for optimising treatment strategies and identifying potential biomarkers. Investigation into the role of tumour microenvironment modulation by SDT is also required to understand the mechanisms underlying the promotion of systemic antitumour immunity. While PDT has shown an abscopal effect in human trials, SDT human clinical trials are only in their early phases and focussed on GBM and DIPG. The results of these are much awaited to evaluate the safety and efficacy of SDT. Future human trials targeting metastatic diseases, such as breast or pancreatic cancer, could hold the potential to translate SDT-induced abscopal and vaccine effects into clinical reality.

## References

[1] Foglietta F, Gola G, Biasibetti E, Capucchio MT, Bruni I, Francovich A, et al. 5-aminolevulinic acid triggered by ultrasound halts tumor proliferation in a syngeneic model of breast cancer. Pharmaceuticals. 2021;14:972. 34681196 10.3390/ph14100972 PMC8540919

[2] Lafond M, Yoshizawa S, Umemura S. Sonodynamic therapy: advances and challenges in clinical translation. J Ultrasound Med. 2019;38:567–80. 30338863 10.1002/jum.14733

[3] Keenlyside A, Marples T, Gao Z, Hu H, Nicely LG, Nogales J, et al. Development and optimisation of in vitro sonodynamic therapy for glioblastoma. Sci Rep. 2023;13:20215. 37980454 10.1038/s41598-023-47562-2 PMC10657375

[4] Rosenthal I, Sostaric JZ, Riesz P. Sonodynamic therapy—a review of the synergistic effects of drugs and ultrasound. Ultrason Sonochem. 2004;11:349–63. 15302020 10.1016/j.ultsonch.2004.03.004

[5] Borah BM, Cacaccio J, Durrani FA, Bshara W, Turowski SG, Spernyak JA, et al. Sonodynamic therapy in combination with photodynamic therapy shows enhanced long-term cure of brain tumor. Sci Rep. 2020;10:21791. 33311561 10.1038/s41598-020-78153-0 PMC7732989

[6] Nabrinsky E, Macklis J, Bitran J. A review of the abscopal effect in the era of immunotherapy. Cureus. 2022;14:e29620. 36321062 10.7759/cureus.29620 PMC9604762

[7] Demaria S, Ng B, Devitt ML, Babb JS, Kawashima N, Liebes L, et al. Ionizing radiation inhibition of distant untreated tumors (abscopal effect) is immune mediated. Int J Radiat Oncol Biol Phys. 2004;58:862–70. 14967443 10.1016/j.ijrobp.2003.09.012

[8] Kessel D. Photodynamic therapy: a brief history. J Clin Med. 2019;8:1581. 31581613 10.3390/jcm8101581 PMC6832404

[9] Niedre M, Patterson MS, Wilson BC. Direct near-infrared luminescence detection of singlet oxygen generated by photodynamic therapy in cells in vitro and tissues in vivo. Photochem Photobio. 2002;75:382–91. 10.1562/0031-8655(2002)075<0382:DNILDO>2.0.CO;2 12003128

[10] Mroz P, Yaroslavsky A, Kharkwal GB, Hamblin MR. Cell death pathways in photodynamic therapy of cancer. Cancers (Basel). 2011;3:2516–39. 23914299 10.3390/cancers3022516 PMC3729395

[11] Inglut CT, Gaitan B, Najafali D, Lopez IA, Connolly NP, Orsila S, et al. Predictors and limitations of the penetration depth of photodynamic effects in the rodent brain. Photochem Photobio. 2020;96:301–9. 10.1111/php.13155 PMC7035972 31441057

[12] Mchale AP, Callan JF, Nomikou N, Fowley C, Callan B. Sonodynamic Therapy: Concept, Mechanism and Application to Cancer Treatment. Adv Exp Med Biol, Springer International Publishing; 2016, p. 429–50. 10.1007/978-3-319-22536-4_22. 10.1007/978-3-319-22536-4_22 26486350

[13] Choi V, Rajora MA, Zheng G. Activating drugs with sound: mechanisms behind sonodynamic therapy and the role of nanomedicine. Bioconjug Chem. 2020;31:967–89. 32129984 10.1021/acs.bioconjchem.0c00029

[14] Ibsen S, Schutt E. Microbubble-mediated ultrasound therapy: a review of its potential in cancer treatment. Drug Des Devel Ther. 2013;7:375. 23667309 10.2147/DDDT.S31564 PMC3650568

[15] McEwan C, Kamila S, Owen J, Nesbitt H, Callan B, Borden M, et al. Combined sonodynamic and antimetabolite therapy for the improved treatment of pancreatic cancer using oxygen loaded microbubbles as a delivery vehicle. Biomaterials. 2016;80:20–32. 26702983 10.1016/j.biomaterials.2015.11.033

[16] Foglietta F, Canaparo R, Francovich A, Arena F, Civera S, Cravotto G, et al. Sonodynamic treatment as an innovative bimodal anticancer approach: shock wave-mediated tumor growth inhibition in a syngeneic breast cancer model. Discov Med. 2015;20:197–205. 26562473

[17] Chen B, Zheng R, Liu D, Li B, Lin J, Zhang W. The tumor affinity of chlorin e6 and its sonodynamic effects on non-small cell lung cancer. Ultrason Sonochem. 2013;20:667–73. 23073382 10.1016/j.ultsonch.2012.09.008

[18] Nonaka M, Yamamoto M, Yoshino S, Umemura S-I, Sasaki K, Fukushima T. Sonodynamic therapy consisting of focused ultrasound and a photosensitizer causes a selective antitumor effect in a rat intracranial glioma model. Anticancer Res. 2009;29:943–50. 19414331

[19] McKaig T, Logan K, Nesbitt H, Callan B, McKeown S, O’Sullivan JM, et al. Ultrasound targeted microbubble destruction using docetaxel and Rose Bengal loaded Microbubbles for targeted Chemo-Sonodynamic therapy treatment of prostate cancer. Eur J Pharmaceutics Biopharmaceutics. 2023;192:196–205. 10.1016/j.ejpb.2023.10.012 37858804

[20] Zhang W, Han B, Gao C, Liu X, Peng Y, Gong C, et al. Integrated platform of oxygen self-enriched nanovesicles: SP94 peptide-directed chemo/sonodynamic therapy for liver cancer. Eur J Pharmaceutics Biopharmaceutics. 2022;179:206–20. 10.1016/j.ejpb.2022.09.012 36150614

[21] Riesz P, Kondo T. Free radical formation induced by ultrasound and its biological implications. Free Radic Biol Med. 1992;13:247–70. 1324205 10.1016/0891-5849(92)90021-8

[22] Fan Z, Kumon RE, Deng CX. Mechanisms of microbubble-facilitated sonoporation for drug and gene delivery. Ther Deliv. 2014;5:467–86. 24856171 10.4155/tde.14.10 PMC4116608

[23] Beguin E, Shrivastava S, Dezhkunov NV, Mchale AP, Callan JF, Stride E. Direct evidence of multibubble sonoluminescence using therapeutic ultrasound and microbubbles. ACS Appl Mater Interfaces. 2019;11:19913–9. 31074968 10.1021/acsami.9b07084 PMC7006998

[24] Pitt WG, Husseini GA, Staples BJ. Ultrasonic drug delivery – a general review. Expert Opin Drug Deliv. 2004;1:37–56. 16296719 10.1517/17425247.1.1.37 PMC1361256

[25] MIŠÍK V, RIESZ P. Free radical intermediates in sonodynamic therapy. Ann N. Y Acad Sci. 2000;899:335–48. 10863551 10.1111/j.1749-6632.2000.tb06198.x

[26] Zhou H, Chen Y, Li P, He X, Zhong J, Hu Z, et al. Sonodynamic therapy for breast cancer: a literature review. Open Chem. 2022;20:1045–56.

[27] Redza-Dutordoir M, Averill-Bates DA. Activation of apoptosis signalling pathways by reactive oxygen species. Biochimica et Biophysica Acta (BBA). Mol Cell Res. 2016;1863:2977–92. 10.1016/j.bbamcr.2016.09.012 27646922

[28] Hu C, Hou B, Xie S. Application of nanosonosensitizer materials in cancer sono-dynamic therapy. RSC Adv. 2022;12:22722–47. 36105955 10.1039/d2ra03786f PMC9376763

[29] Xing X, Zhao S, Xu T, Huang L, Zhang Y, Lan M, et al. Advances and perspectives in organic sonosensitizers for sonodynamic therapy. Coord Chem Rev. 2021;445:214087.

[30] Cao X, Li M, Liu Q, Zhao J, Lu X, Wang J. Inorganic sonosensitizers for sonodynamic therapy in cancer treatment. Small. 2023;19:e2303195 10.1002/smll.202303195. 37323087 10.1002/smll.202303195

[31] Deng X, Shao Z, Zhao Y. Development of porphyrin and titanium dioxide sonosensitizers for sonodynamic cancer therapy. Biomater Transl. 2021;2:72–85. 35837259 10.3877/cma.j.issn.2096-112X.2021.01.009 PMC9255825

[32] Wachowska M, Muchowicz A, Firczuk M, Gabrysiak M, Winiarska M, Wańczyk M, et al. Aminolevulinic acid (ALA) as a prodrug in photodynamic therapy of cancer. Molecules. 2011;16:4140–64.

[33] Raspagliesi L, D’Ammando A, Gionso M, Sheybani ND, Lopes M-B, Moore D, et al. Intracranial sonodynamic therapy with 5-aminolevulinic acid and sodium fluorescein: safety study in a porcine model. Front Oncol. 2021;11:679989. 34235081 10.3389/fonc.2021.679989 PMC8256685

[34] Suero Molina E, Stögbauer L, Jeibmann A, Warneke N, Stummer W. Validating a new generation filter system for visualizing 5-ALA-induced PpIX fluorescence in malignant glioma surgery: a proof of principle study. Acta Neurochir (Wien). 2020;162:785–93. 32034493 10.1007/s00701-020-04227-7 PMC7066295

[35] Li Y, Zhou Q, Hu Z, Yang B, Li Q, Wang J, et al. 5-aminolevulinic acid-based sonodynamic therapy induces the apoptosis of osteosarcoma in mice. PLoS One. 2015;10:e0132074. 26161801 10.1371/journal.pone.0132074 PMC4498784

[36] Ohgari Y, Nakayasu Y, Kitajima S, Sawamoto M, Mori H, Shimokawa O, et al. Mechanisms involved in δ-aminolevulinic acid (ALA)-induced photosensitivity of tumor cells: relation of ferrochelatase and uptake of ALA to the accumulation of protoporphyrin. Biochem Pharm. 2005;71:42–9. 16288996 10.1016/j.bcp.2005.10.019

[37] Nasir-Moin M, Wadiura LI, Sacalean V, Juros D, Movahed-Ezazi M, Lock EK, et al. Localization of protoporphyrin IX during glioma-resection surgery via paired stimulated Raman histology and fluorescence microscopy. Nat Biomed Eng. 2024;8:672–88. 38987630 10.1038/s41551-024-01217-3

[38] Vanerio N, Stijnen M, de Mol BAJM, Kock LM. Biomedical applications of photo- and sono-activated rose Bengal: a review. Photobiomodul Photomed Laser Surg. 2019;37:383–94. 31180251 10.1089/photob.2018.4604

[39] Ge J, Lan M, Zhou B, Liu W, Guo L, Wang H, et al. A graphene quantum dot photodynamic therapy agent with high singlet oxygen generation. Nat Commun. 2014;5:4596. 25105845 10.1038/ncomms5596 PMC4143951

[40] Robinson JT, Tabakman SM, Liang Y, Wang H, Sanchez Casalongue H, Vinh D, et al. Ultrasmall reduced graphene oxide with high near-infrared absorbance for photothermal therapy. J Am Chem Soc. 2011;133:6825–31. 21476500 10.1021/ja2010175

[41] Mandal AA, Kushwaha R, Yadav AK, Banerjee S. Metal complexes for cancer sonodynamic therapy. ChemBioChem. 2023;24:e202200597 10.1002/cbic.202200597. 36385722 10.1002/cbic.202200597

[42] Zhang M, Shao S, Yue H, Wang X, Zhang W, Chen F, et al. High stability Au NPs: from design to application in nanomedicine. Int J Nanomed. 2021;16:6067–94. 10.2147/IJN.S322900 PMC8418318 34511906

[43] Chen P, Zhang P, Shah NH, Cui Y, Wang Y. A comprehensive review of inorganic sonosensitizers for sonodynamic therapy. Int J Mol Sci. 2023;24:12001 10.3390/ijms241512001. 37569377 10.3390/ijms241512001 PMC10418994

[44] Osminkina LA, Sivakov VA, Mysov GA, Georgobiani VA, Natashina UА, Talkenberg F, et al. Nanoparticles prepared from porous silicon nanowires for bio-imaging and sonodynamic therapy. Nanoscale Res Lett. 2014;9:463. 25288909 10.1186/1556-276X-9-463 PMC4185383

[45] Mole RH. Whole body irradiation—radiobiology or medicine? Br J Radio. 1953;26:234–41. 10.1259/0007-1285-26-305-234 13042090

[46] Janopaul-Naylor JR, Shen Y, Qian DC, Buchwald ZS. The abscopal effect: a review of pre-clinical and clinical advances. Int J Mol Sci. 2021;22:11061. 34681719 10.3390/ijms222011061 PMC8537037

[47] Nesbitt H, Logan K, Thomas K, Callan B, Gao J, Mckaig T, et al. Sonodynamic therapy complements PD-L1 immune checkpoint inhibition in a murine model of pancreatic cancer. Cancer Lett. 2021;517:88–95. 34119606 10.1016/j.canlet.2021.06.003

[48] Rodriguez-Ruiz ME, Rodriguez I, Leaman O, López-Campos F, Montero A, Conde AJ, et al. Immune mechanisms mediating abscopal effects in radioimmunotherapy. Pharm Ther. 2019;196:195–203. 10.1016/j.pharmthera.2018.12.002 30529041

[49] Grass GD, Krishna N, Kim S. The immune mechanisms of abscopal effect in radiation therapy. Curr Probl Cancer. 2016;40:10–24. 26612692 10.1016/j.currproblcancer.2015.10.003

[50] Kroemer G, Galluzzi L, Kepp O, Zitvogel L. Immunogenic cell death in cancer therapy. Annu Rev Immunol. 2013;31:51–72. 23157435 10.1146/annurev-immunol-032712-100008

[51] Galluzzi L, Buqué A, Kepp O, Zitvogel L, Kroemer G. Immunogenic cell death in cancer and infectious disease. Nat Rev Immunol. 2017;17:97–111. 27748397 10.1038/nri.2016.107

[52] Krysko DV, Garg AD, Kaczmarek A, Krysko O, Agostinis P, Vandenabeele P. Immunogenic cell death and DAMPs in cancer therapy. Nat Rev Cancer. 2012;12:860–75. 23151605 10.1038/nrc3380

[53] Muenst S, Läubli H, Soysal SD, Zippelius A, Tzankov A, Hoeller S. The immune system and cancer evasion strategies: therapeutic concepts. J Intern Med. 2016;279:541–62. 26748421 10.1111/joim.12470

[54] Bhatia A, Kumar Y. Cellular and molecular mechanisms in cancer immune escape: a comprehensive review. Expert Rev Clin Immunol. 2014;10:41–62. 24325346 10.1586/1744666X.2014.865519

[55] Garrido F, Aptsiauri N. Cancer immune escape: MHC expression in primary tumours versus metastases. Immunology. 2019;158:255–66. 31509607 10.1111/imm.13114 PMC6856929

[56] Xi Y, Chen L, Tang J, Yu B, Shen W, Niu X. Amplifying “eat me signal” by immunogenic cell death for potentiating cancer immunotherapy. Immunol Rev. 2024;321:94–114. 37550950 10.1111/imr.13251

[57] Obeid M, Tesniere A, Ghiringhelli F, Fimia GM, Apetoh L, Perfettini J-L, et al. Calreticulin exposure dictates the immunogenicity of cancer cell death. Nat Med. 2007;13:54–61. 17187072 10.1038/nm1523

[58] Ghiringhelli F, Apetoh L, Tesniere A, Aymeric L, Ma Y, Ortiz C, et al. Activation of the NLRP3 inflammasome in dendritic cells induces IL-1β–dependent adaptive immunity against tumors. Nat Med. 2009;15:1170–8. 19767732 10.1038/nm.2028

[59] Ahmed A, Tait SWG. Targeting immunogenic cell death in cancer. Mol Oncol. 2020;14:2994–3006. 33179413 10.1002/1878-0261.12851 PMC7718954

[60] Rovere‐Querini P, Capobianco A, Scaffidi P, Valentinis B, Catalanotti F, Giazzon M, et al. HMGB1 is an endogenous immune adjuvant released by necrotic cells. EMBO Rep. 2004;5:825–30. 15272298 10.1038/sj.embor.7400205 PMC1299116

[61] Rodríguez ME, Cogno IS, Sanabria LSM, Morán YS, Rivarola VA. Heat shock proteins in the context of photodynamic therapy: autophagy, apoptosis and immunogenic cell death. Photochem Photobiol Sci. 2016;15:1090–102. 27471925 10.1039/c6pp00097e

[62] Troitskaya OS, Novak DD, Richter VA, Koval OA. Immunogenic cell death in cancer therapy. Acta Nat. 2022;14:40–53. 10.32607/actanaturae.11523 PMC9013441 35441043

[63] Truxova I, Hensler M, Skapa P, Halaska MJ, Laco J, Ryska A, et al. Chapter Three - Rationale for the Combination of Dendritic Cell-Based Vaccination Approaches With Chemotherapy Agents. In: Galluzzi L, editor. Int Rev Cell Mol Biol. 330, Academic Press; 2017, p. 115–56. 10.1016/bs.ircmb.2016.09.003 28215530

[64] Lamberti MJ, Nigro A, Mentucci FM, Rumie Vittar NB, Casolaro V, Dal Col J. Dendritic cells and immunogenic cancer cell death: a combination for improving antitumor immunity. Pharmaceutics. 2020;12:256. 32178288 10.3390/pharmaceutics12030256 PMC7151083

[65] Magee CN, Boenisch O, Najafian N. The role of costimulatory molecules in directing the functional differentiation of alloreactive T helper cells. Am J Transplant. 2012;12:2588–600. 22759274 10.1111/j.1600-6143.2012.04180.x PMC3459149

[66] Dalod M, Chelbi R, Malissen B, Lawrence T. Dendritic cell maturation: functional specialization through signaling specificity and transcriptional programming. EMBO J. 2014;33:1104–16. 24737868 10.1002/embj.201488027 PMC4193918

[67] Dudek AM, Martin S, Garg AD, Agostinis P. Immature, semi-mature, and fully mature dendritic cells: toward a DC-cancer cells interface that augments anticancer immunity. Front Immunol. 2013;4:438. 24376443 10.3389/fimmu.2013.00438 PMC3858649

[68] Adams NM, Grassmann S, Sun JC. Clonal expansion of innate and adaptive lymphocytes. Nat Rev Immunol. 2020;20:694–707. 32424244 10.1038/s41577-020-0307-4 PMC13119617

[69] Barry M, Bleackley RC. Cytotoxic T lymphocytes: all roads lead to death. Nat Rev Immunol. 2002;2:401–9. 12093006 10.1038/nri819

[70] Voskoboinik I, Dunstone MA, Baran K, Whisstock JC, Trapani JA. Perforin: structure, function, and role in human immunopathology. Immunol Rev. 2010;235:35–54. 20536554 10.1111/j.0105-2896.2010.00896.x

[71] Montinaro A, Walczak H. Harnessing TRAIL-induced cell death for cancer therapy: a long walk with thrilling discoveries. Cell Death Differ. 2023;30:237–49. 36195672 10.1038/s41418-022-01059-z PMC9950482

[72] Mercogliano MF, Bruni S, Mauro F, Elizalde PV, Schillaci R. Harnessing tumor necrosis factor alpha to achieve effective cancer immunotherapy. Cancers. 2021;13:564. 33540543 10.3390/cancers13030564 PMC7985780

[73] Rahman M, Sawyer WG, Lindhorst S, Deleyrolle LP, Harrison JK, Karachi A, et al. Adult immuno-oncology: using past failures to inform the future. Neuro Oncol. 2020;22:1249–61. 32391559 10.1093/neuonc/noaa116 PMC7523444

[74] Thorsson V, Gibbs DL, Brown SD, Wolf D, Bortone DS, Ou Yang T-H, et al. The immune landscape of cancer. Immunity. 2018;48:812–830.e14. 29628290 10.1016/j.immuni.2018.03.023 PMC5982584

[75] Frederico SC, Hancock JC, Brettschneider EES, Ratnam NM, Gilbert MR, Terabe M. Making a cold tumor hot: the role of vaccines in the treatment of glioblastoma. Front Oncol. 2021;11:672508. 34041034 10.3389/fonc.2021.672508 PMC8141615

[76] Galon J, Bruni D. Approaches to treat immune hot, altered and cold tumours with combination immunotherapies. Nat Rev Drug Discov. 2019;18:197–218. 30610226 10.1038/s41573-018-0007-y

[77] Galon J, Costes A, Sanchez-Cabo F, Kirilovsky A, Mlecnik B, Lagorce-Pagès C, et al. Type, density, and location of immune cells within human colorectal tumors predict clinical outcome. Science. 1979;2006:1960–4. 10.1126/science.1129139 17008531

[78] Luksik AS, Yazigi E, Shah P, Jackson CM. CAR T cell therapy in glioblastoma: overcoming challenges related to antigen expression. Cancers. 2023;15:1414. 36900205 10.3390/cancers15051414 PMC10000604

[79] Burster T, Gärtner F, Bulach C, Zhanapiya A, Gihring A, Knippschild U. Regulation of MHC I molecules in glioblastoma cells and the sensitizing of NK cells. Pharmaceuticals. 2021;14:236. 33800301 10.3390/ph14030236 PMC7998501

[80] Segura-Collar B, Hiller-Vallina S, de Dios O, Caamaño-Moreno M, Mondejar-Ruescas L, Sepulveda-Sanchez JM, et al. Advanced immunotherapies for glioblastoma: tumor neoantigen vaccines in combination with immunomodulators. Acta Neuropathol Commun. 2023;11:79. 37165457 10.1186/s40478-023-01569-y PMC10171733

[81] Quail DF, Joyce JA. The microenvironmental landscape of brain tumors. Cancer Cell. 2017;31:326–41. 28292436 10.1016/j.ccell.2017.02.009 PMC5424263

[82] Bausart M, Préat V, Malfanti A. Immunotherapy for glioblastoma: the promise of combination strategies. J Exp Clin Cancer Res. 2022;41:35. 35078492 10.1186/s13046-022-02251-2 PMC8787896

[83] Munegowda MA, Fisher C, Molehuis D, Foltz W, Roufaiel M, Bassan J, et al. Efficacy of ruthenium coordination complex–based Rutherrin in a preclinical rat glioblastoma model. Neurooncol Adv. 2019;1:vdz006. 32642649 10.1093/noajnl/vdz006 PMC7212850

[84] Yang H, Tu L, Li J, Bai S, Hu Z, Yin P, et al. Deep and precise lighting-up/combat diseases through sonodynamic agents integrating molecular imaging and therapy modalities. Coord Chem Rev. 2022;453:214333.

[85] Bhanja D, Wilding H, Baroz A, Trifoi M, Shenoy G, Slagle-Webb B, et al. Photodynamic therapy for glioblastoma: illuminating the path toward clinical applicability. Cancers. 2023;15:3427. 37444537 10.3390/cancers15133427 PMC10341187

[86] Koshy M, Villano JL, Dolecek TA, Howard A, Mahmood U, Chmura SJ, et al. Improved survival time trends for glioblastoma using the SEER 17 population-based registries. J Neurooncol 2012;107:207–12. 10.1007/s11060-011-0738-7 PMC4077033 21984115

[87] Prada F, Sheybani N, Franzini A, Moore D, Cordeiro D, Sheehan J, et al. Fluorescein-mediated sonodynamic therapy in a rat glioma model. J Neurooncol. 2020;148:445–54. 32500440 10.1007/s11060-020-03536-2

[88] Syed HR, Patel N, Kilburn LB, Fonseca A, Stabingas K, Clanton R, et al. Phase 1/2, first-in-child study of sonodynamic therapy (SDT) using low intensity focused ultrasound and 5-aminolevulinic acid (ALA) for patients with diffuse intrinsic pontine glioma. Journal of Clinical Oncology 2023;41:TPS10070–TPS10070.

[89] Gwak H-S, Park HJ. Developing chemotherapy for diffuse pontine intrinsic gliomas (DIPG). Crit Rev Oncol Hematol. 2017;120:111–9. 29198324 10.1016/j.critrevonc.2017.10.013

[90] Vanan MI, Eisenstat DD. DIPG in children – what can we learn from the past? Front Oncol. 2015;5:237. 26557503 10.3389/fonc.2015.00237 PMC4617108

[91] Zhang Q, Bao C, Cai X, Jin L, Sun L, Lang Y, et al. Sonodynamic therapy‐assisted immunotherapy: A novel modality for cancer treatment. Cancer Sci. 2018;109:1330–45. 29575297 10.1111/cas.13578 PMC5980136

[92] Wang Y, Gong F, Han Z, Lei H, Zhou Y, Cheng S, et al. Oxygen‐deficient molybdenum oxide nanosensitizers for ultrasound‐enhanced cancer metalloimmunotherapy. Angew Chem Int Ed. 2023;62:e202215467. 10.1002/anie.202215467 36591974

[93] Nicholas D, Nesbitt H, Farrell S, Logan K, Mcmullin E, Gillan T, et al. Exploiting a Rose Bengal-bearing, oxygen-producing nanoparticle for SDT and associated immune-mediated therapeutic effects in the treatment of pancreatic cancer. Eur J Pharmaceutics Biopharmaceutics. 2021;163:49–59. 10.1016/j.ejpb.2021.03.005 33798727

[94] Qiao K, Luo C, Huang R, Xiang J, Pan Y, Zhang S, et al. Ultrasound triggered tumor metabolism suppressor induces tumor starvation for enhanced sonodynamic immunotherapy of breast cancer. Int J Nanomed. 2023;18:3801–11. 10.2147/IJN.S413543 PMC10349352 37457803

[95] Chen H, Liu L, Ma A, Yin T, Chen Z, Liang R, et al. Noninvasively immunogenic sonodynamic therapy with manganese protoporphyrin liposomes against triple-negative breast cancer. Biomaterials. 2021;269:120639. 33434714 10.1016/j.biomaterials.2020.120639

[96] Hadi MM, Farrell S, Nesbitt H, Thomas K, Kubajewska I, Ng A, et al. Nanotechnology-augmented sonodynamic therapy and associated immune-mediated effects for the treatment of pancreatic ductal adenocarcinoma. J Cancer Res Clin Oncol. 2023;149:5007–23. 36319895 10.1007/s00432-022-04418-y PMC10349707

[97] Zhan G, Xu Q, Zhang Z, Wei Z, Yong T, Bie N, et al. Biomimetic sonodynamic therapy-nanovaccine integration platform potentiates Anti-PD-1 therapy in hypoxic tumors. Nano Today. 2021;38:101195.

[98] Yang Y, Ge J, Li G, Lei H, Chen L, Gong Y, et al. Manganese-doping titanium disulfide cascade nanobioreactors for sequential gas-sonodynamic strategy with immune checkpoint blockade therapy of cancer. Nano Today 2022;46:101585.

[99] Fu S, Yang R, Ren J, Liu J, Zhang L, Xu Z, et al. Catalytically active CoFe2O4 nanoflowers for augmented sonodynamic and chemodynamic combination therapy with elicitation of robust immune response. ACS Nano. 2021;15:11953–69. 34142808 10.1021/acsnano.1c03128

[100] Zheng J, Sun J, Chen J, Zhu S, Chen S, Liu Y, et al. Oxygen and oxaliplatin-loaded nanoparticles combined with photo-sonodynamic inducing enhanced immunogenic cell death in syngeneic mouse models of ovarian cancer. J Controlled Release. 2021;332:448–59. 10.1016/j.jconrel.2021.02.032 33662456

[101] Letchuman V, Ampie L, Shah AH, Brown DA, Heiss JD, Chittiboina P. Syngeneic murine glioblastoma models: reactionary immune changes and immunotherapy intervention outcomes. Neurosurg Focus. 2022;52:E5. 35104794 10.3171/2021.11.FOCUS21556 PMC10851929

[102] Stepp H, Stummer W. 5‐ALA in the management of malignant glioma. Lasers Surg Med. 2018;50:399–419. 29737540 10.1002/lsm.22933

[103] Moloudi K, Sarbadhikary P, Abrahamse H, George BP. Understanding the photodynamic therapy induced bystander and abscopal effects: a review. Antioxidants. 2023;12:1434. 37507972 10.3390/antiox12071434 PMC10376621

[104] Lou J, Aragaki M, Bernards N, Chee T, Gregor A, Hiraishi Y, et al. Repeated photodynamic therapy mediates the abscopal effect through multiple innate and adaptive immune responses with and without immune checkpoint therapy. Biomaterials. 2023;292:121918. 36442438 10.1016/j.biomaterials.2022.121918

[105] Stummer W, Beck T, Beyer W, Mehrkens JH, Obermeier A, Etminan N, et al. Long-sustaining response in a patient with non-resectable, distant recurrence of glioblastoma multiforme treated by interstitial photodynamic therapy using 5-ALA: case report. J Neurooncol. 2008;87:103–9. 18034212 10.1007/s11060-007-9497-x

[106] Marcus SL, de Souza MP. Theranostic uses of the heme pathway in neuro-oncology: protoporphyrin IX (PpIX) and its journey from photodynamic therapy (PDT) through photodynamic diagnosis (PDD) to sonodynamic therapy (SDT). Cancers. 2024;16:740. 38398131 10.3390/cancers16040740 PMC10886505

[107] Sanai N, Tien A-C, Tovmasyan A, Chang Y-W, Margaryan T, Hendrickson K, et al. CTNI-13. a first-in-human phase 0/1 trial of 5-aminolevulinic acid sonodynamic theraPY (5-ALA SDT) in recurrent glioblastoma. Neuro Oncol. 2022;24:vii72–3.

[108] Izadifar Z, Izadifar Z, Chapman D, Babyn P. An introduction to high intensity focused ultrasound: systematic review on principles, devices, and clinical applications. J Clin Med. 2020;9:460. 32046072 10.3390/jcm9020460 PMC7073974

[109] Yamamoto K, Sarica C, Loh A, Vetkas A, Samuel N, Milano V, et al. Magnetic resonance-guided focused ultrasound for the treatment of tremor. Expert Rev Neurother. 2022;22:849–61. 36469578 10.1080/14737175.2022.2147826

[110] Wu F, Zhou L, Chen WR. Host antitumour immune responses to HIFU ablation. Int J Hyperth. 2007;23:165–71. 10.1080/02656730701206638 17578340

[111] Eranki A, Srinivasan P, Ries M, Kim A, Lazarski CA, Rossi CT, et al. High-intensity focused ultrasound (HIFU) triggers immune sensitization of refractory murine neuroblastoma to checkpoint inhibitor therapy. Clin Cancer Res. 2020;26:1152–61. 31615935 10.1158/1078-0432.CCR-19-1604 PMC9009723

[112] Pepple AL, Guy JL, McGinnis R, Felsted AE, Song B, Hubbard R, et al. Spatiotemporal local and abscopal cell death and immune responses to histotripsy focused ultrasound tumor ablation. Front Immunol. 2023;14:1012799 10.3389/fimmu.2023.1012799. 36756111 10.3389/fimmu.2023.1012799 PMC9900174

